# Physiological and pathological impact of AQP1 knockout in mice

**DOI:** 10.1042/BSR20182303

**Published:** 2019-05-14

**Authors:** Ying Hua, Xinxin Ying, Yiyu Qian, Haibin Liu, Yehui Lan, Ailan Xie, Xueqiong Zhu

**Affiliations:** 1 Department of Obstetrics and Gynecology, The Second Affiliated Hospital of Wenzhou Medical University, Wenzhou 325027, China; 2 Department of Hepatobiliary Surgery, The Second Affiliated Hospital of Wenzhou Medical University, Wenzhou 325027, China

**Keywords:** Aquaporin 1, Knockout, Physiological Change, Pathological Change

## Abstract

Aquaporin 1 (AQP1) is a glycoprotein responsible for water passive transport quickly across biological membrane. Here, we reviewed the structural and functional impacts of AQP1 knockout (AQP1-KO) in animal or cell culture models. AQP1 gene deletion can cause a large number of abnormalities including the disturbance in epithelial fluid secretion, polyhydramnios, deficiency of urinary concentrating function, and impairment of pain perception. AQP1-KO mice also displayed aberrations of cardiovascular, gastrointestinal and hepatobiliary, and kidney functions as well as placenta and embryo development. Moreover, AQP1-KO perturbed tumor angiogenesis and led to reduced brain injury upon trauma. On the cellular level, AQP1-KO caused neuroinflammation, aberrant cell proliferation and migration, and macrophages infiltration. Mechanistic studies confirmed that AQP1 gene products regulate the secretory function and participated in balancing the osmotic water flux across the peritoneal membrane. The available data indicated that AQP1 might serve as a potential target for developing novel therapeutic approaches against diverse human diseases.

## Introduction

Aquaporins (AQPs) are a family of small, integral membrane proteins that facilitate water transport and small neutral solutes across a variety of biological membranes. Aquaporin 1 (AQP1) is the first identified member of the AQP family and is a water-selective transporting protein [1]. The phenotype analysis of transgenic mice deficient in AQP1 has provided new insights into the role of AQP1 in the functions of kidney, lung, placenta, peritoneum, eye, gland secretion, heart, and cells [2–64]. AQP1 deletion is associated with a variety of abnormalities including polyuria, polyhydramnios, cataract, cardiovascular homeostasis disorder, angiogenesis anomaly, pain perception impairment, and neuroinflammation. In addition, AQP1 deletion results in protection of brain trauma and a decreased tumor growth. The main goal of this review is to update and summarize the knowledge from AQP1-KO animal models or cells, and current understanding on the role of AQP1 in organ physiology under normal and pathological conditions.

### Effect of aquaporin-1 gene knockout on red blood cells

AQP1 is the major water channel of human red blood cells. Some studies revealed that the AQP1 regulated water permeability in red cells. Mathai *et al.* [2] found that even though hematologic consequences of total AQP1 deficiency were not severe and the AQP1 knocked out (Colton-null) red cells had normal morphology, normal hematocrit, and normal hemoglobin levels, these cells exhibited slightly shortened life span, a reduced membrane surface area, and a dramatically reduced osmotic water permeability [2] [Table 1]. There was no difference in glycerol transport between the normal and Colton-null red cells. However, the presence of functional AQP3 in the human red cell membrane may account for the glycerol permeability of these cells and the residual water permeability of AQP1-deficient erythrocytes [3]. Interestingly, erythrocyte water permeability was remarkably reduced by AQP1 deletion but not further reduced by the deletion of AQP1 and AQP3 [4]. In addition, the AQP1/urea transporter UT-B double-knockout mice had reduced survival, retarded growth, defective urinary concentrating ability and a 4.2-fold reduction in the osmotic water permeability in erythrocytes compared with single knockout mice deficient in AQP1. But erythrocyte size and morphology were not affected [5]. Taken together, these studies [2–5] provided insight into the physiological consequences of red cell membrane water permeability.

**Table 1:** Main effect of AQP1-KO on organs and system

Target cell/tissue	Outcome	References
Red blood cell	• Slightly shortened red cell life span, reduced membrane surface area, and a dramatically reduced osmotic water permeability	[2]
Kidney	• Inability to generate a hypertonic medullary interstitium by countercurrent multiplication	[4,8–13,18,20]
	• Impairment in the migration of proximal tubule cells and cell proliferation after acute kiney injury	[14,15]
	• Lower glomerular filtration rate (GFR) and renal blood flow	[16]
Brain	• Protective in a model of brain trauma	[22–26]
	• Reduced thermal inflammatory pain perception evoked by bradykinin, prostaglandin E2, and capsaicin as well as reduced cold pain perception and distinct electrophysiological defects	[27–31]
The lungs	• Changed permeability in different degree in different part	[32,33]
Placenta	• Lower number of embryos, lower fetal weight	[37–39]
Fetal membranes	• Increased amniotic fluid volume and reduced osmolality	[40,41]
Peritoneum	• Significantly reduced osmotically induced water movement, strongly decreased indices for AQP1-related transcellular water transport	[42–44]
Eye	• Reduced corneal thickness, reduces osmotic water permeability across the corneal endothelium, impaired keratocyte migration	[45,46]
	• Accelerated cataract formation	[47,48]
Gland secretion	• Reduced prohormone convertase 1/3, carboxypeptidase E, attenuated regulated secretion of ACTH, decreased dense-core secretory granule (DCSG) proteins biogenesis	[49]
Cardiovascular system	• Marked microcardia, decreased myocyte transverse dimensions and a significant decrease in the thickness of the arterial walls both in the aorta and mesenteric artery	[54]
	• Promoted atherosclerosis	[55]
Digestive system	• Acquired an oily appearance, manifested serum hypotriglyceridemia and developed steatorrhea with increased stool triglyceride content, and greater lipase activity	[58]
Cellular level	• Reduced angiogenesis, impaired cell migration, abnormal vessel formation, and abnormal microvascular anatomy	[61,63,64]
	• Reduced relative plasma membrane water permeability in chondrocyte	[62]

It is noteworthy that a study by Endeward *et al.* [6] provided evidence for a potential role of AQP1 proteins in gas transportation in red cell [6]. PCO_2_ were significantly reduced (by ∼60%) in aquaporin-1-deficient cells than in normal red cells, while PH_CO3–_ is identical in both types of red cells, indicating that AQP1 may contribute to CO_2_ permeation across the membrane. However, Ripoche *et al.* [7] reported that AQP1-KO has no significant influence on CO_2_ transport in red cells. Instead, red cells of AQP1^−/−^ mice exhibited a significantly reduced transport rate of NH_3_ [7]. Thus, these results suggest that AQP1 does not merely serve as a major water channel, but may also assistant gas transportation across the human erythrocyte membrane. Further studies will be necessary to clarify the mechanism by which AQP1 is a major pathway for gas transport across the human erythrocyte membrane.

### Effect of aquaporin-1 gene knockout on kidney

Knockout technology approaches provided pivotal information on the pathophysiological role of AQP1 in kidney [8–21] [Table 1]. Transgenic mice lacking AQP1 suffered an 8-fold reduction in water permeability in proximal tubule membrane vesicles, resulting in disability to concentrate urine in response to water deprivation or desmopressin (DDAVP) administration [8]. AQP1 deletion led to a 78% decrease in osmotic water permeability across the proximal tubule epithelium whereas net fluid reabsorption both *in vitro* and *in vivo* was reduced by only approximately 50%, which suggested that AQP1 deletion affected spontaneous, actively driven fluid reabsorption from the lumen and luminal hypotonicity must be greater in the proximal tubules of the AQP1 knockout (AQP1-KO) mice compared with wild-type (WT) mice [9]. The results were confirmed in the subsequent study showing AQP1 deficiency in mice generated marked luminal hypotonicity in proximal tubules compatible with the retrieval of a hypertonic absorbate and indicating that near-isosmolar fluid absorption requires functional AQP1 [10]. In view of the decrease in transepithelial water permeability and the decrease in intramembrane particles (IMP) density in thin descending limb of Henle (TDLH) of AQP1-deficient mice, the changes in TDLH were primarily responsible for the urinary defect in concentrating efficiency [11]. Taken together, these studies [8–11] suggest that the primary renal defect in AQP1-KO mice is the inability to generate a hypertonic medullary interstitium by countercurrent multiplication [Figure 2].

**Figure 1:**
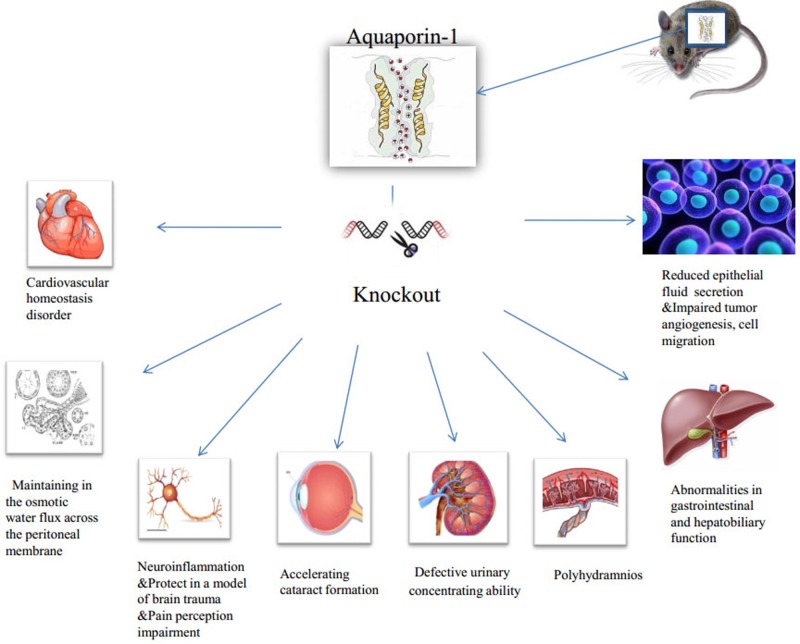
Physiological and pathological impact of AQP1-KO on organs and system In AQP1-KO animal models or their cell culture models, AQP1 deletion was associated with defective urinary concentrating ability, polyhydramnios, cardiovascular homeostasis disorder, accelerating cataract formation, abnormalities in gastrointestinal and hepatobiliary function, neuroinflammation and protect in a model of brain trauma, reduced epithelial fluid secretion and impaired tumor angiogenesis and cell migration, and participating in the osmotic water flux across the peritoneal membrane.

AQP1-deficient mice are unable to generate a hypertonic medullary interstitium, which may play a role in the expression of H^+^-ATPase in inner medullary collecting duct (IMCD) cells [Figure 1]. The expression of H^+^-ATPase in IMCD cells in AQP1 null mice was up-regulated, leading to a decrease in urinary pH [12]. Also, the deletion of AQP1 is associated with increased prevalence of intercalated cells in the IMCD, and the appearance of strong immunoreactivity against —, a marker for the apical plasma membrane of IMCD cells. However, there were no changes in the level of H^+^-ATPase expression in intercalated cells in any segments of the collecting duct, cortex or outer medulla of AQP1 null mice compared with WT mice [12]. The loss of the hyperosmotic renal interstitium drastically altered the renal medullary gene expression profile in AQP1 null mice [13]. The overall pattern of gene expression in the renal medullas of AQP1 null mice was the down-regulation of several heat shock and stress genes, several housekeeping genes, and genes encoding mitochondrial enzymes, Aldose reductase genes that encode the cytochrome c oxidase complex, the F1/F0 ATPase subunits as well as mitochondrial H^+^-ATP synthase F1, Na^+^-K^+^-ATPase β-subunit, adenylate kinase 2 and NADH dehydrogenase [13]. Also, McReynolds *et al.* [13] observed a loss of vasopressin type 2 receptor (V2R) mRNA expression in renal medullas of the AQP1 null mice.

**Figure 2:**
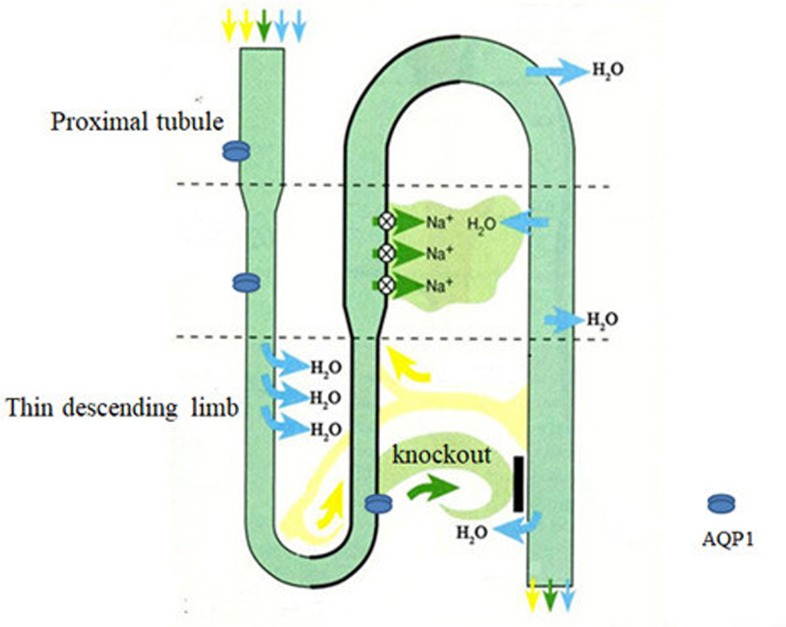
AQP1-KO mice cannot generate a hypertonic medullary interstitium by countercurrent multiplication AQP1-KO mice have suffered an 8-fold reduction in water permeability in proximal tubule membrane vesicles and driven fluid reabsorption from the lumen and luminal hypotonicity greater in the proximal tubules, resulting in disability to concentrate urine.

Distinct from its established role in transepithelial and transendothelial water transport and in the urinary concentrating mechanism, AQP1-deficiency resulted in a significant impairment in the migration of proximal tubule cells *in vitro*, which was corrected by adenovirus-mediated reintroduction of AQP1. A remarkable more severe proximal tubule damage was observed in kidneys of AQP1 null mice after ischemia-reperfusion [14]. Following vasopressin receptor subtype 2 activation, a significant increase in medullary collecting duct cell proliferation, accompanied by changes in expression of cell cycle genes, in AQP1 null mice was confirmed [15]. Thus, AQP1-facilitated cell migration and cell proliferation may be important for the structural and functional regeneration of tubules after acute kidney injury [Table 1].

After endotoxemia, water intake and urinary output in AQP1-KO mice were significantly increased, and urinary osmolality was significantly decreased compared with WT mice. Urinary sodium excretion and fractional sodium excretion were higher in AQP1-KO mice compared with WT mice in endotoxemia, which were accompanied by more severe tubular injury. These responses in the AQP1-KO mice led to lower glomerular filtration rate (GFR) and renal blood flow [16]. Thus, the polyuria in AQP1-KO mice does not appear to protect against endotoxemia-induced acute kidney injury but rather absence of AQP1 predisposed to enhanced endotoxemic renal injury.

To investigate the expression change of other renal aquaporins that might be involved in kidney fluid balance in AQP1-KO, Ma *et al.* [8] found that AQP1 deletion did not change the expression of AQP3 and AQP4, but was associated with a mild increase in the expression of AQP2 transcript and protein in kidney. But no V2R-mediated increase in AQP2 expression occurred in the collecting ducts of these AQP1 null animals [15]. The higher serum osmolality in AQP1-KO mice during endotoxemia in the absence of water repletion was associated with higher AQP2, AQP3, and Na^+^-K^+^-2Cl^−^ cotransporter type 2 expression and a lower Na^+^/H^+^ exchanger type 3 protein expression than that in WT mice during endotoxemia [16] [Table 1]. Furthermore, Morris *et al.* [17] found that there were pronounced changes in the expression levels of the major solute transporter proteins such as a significant decrease in the expression of the UT-A1 (a urea transporter) mRNA and protein as well as a significantly reduced AQP4 (a basolateral water channel) protein levels in the IMCDs of AQP1-KO mice. The urea permeability of the IMCD was significantly reduced in AQP1-KO mice. In contrast, there was an increased expression of three proteins normally expressed at lower levels in IMCD than in the cortical collecting duct (CCD): AQP3 and the epithelial sodium channel subunits β-ENaC and γ-ENaC [17]. Thus, expression patterns of multiple transport proteins known to play prominent roles in the physiology of the IMCD were altered in AQP1-KO mice.

In addition, the deletion of AQP1 and AQP3 together resulted in a lower base-line urine osmolality compared with mice lacking AQP1 [4]. Also, AQP1/AQP7 double-knockout mice and AQP1/AQP4 double-knockout mice had a reduction in urinary concentrating ability compared with AQP1 solo knockout mice [18,19]. Moreover, AQP1/AQP7 double-knockout mice displayed a significantly increased urine excretion accompanied by a proportional decrease in urine osmolality. Taken together, these studies indicated synergistic effects by some aquaporins on liquid exchange in the kidney.

The effect of joint knockout of AQP1 and other factors on renal fluid exchange was also investigated [20,21]. Schnermann *et al.* [20] found that co-deletion of claudin-2 and AQP1 did not deliver a greater impact on proximal tubule fluid reabsorption than AQP1 single deletion [20]. Compared with AQP1 deficient mice, double knockout of AQP1 and A1AR^−/−^ (adenosine 1 receptor) generated by crossing AQP1^−/−^ with A1AR^−/−^ were found to have a reduced proximal tubule fluid absorption and a significantly increased single nephron GFR (SNGFR), while there is a normal GFR. Moreover, tubuloglomerular feedback responses of SNGFR were abolished in AQP1/A1AR^−/−^ mice [21].

In conclusion, much of the information had been derived from studies in AQP1-KO animal models or their cell culture models, which shed insights into the renal pathophysiological function. According to the data obtained from AQP-KO mice studies, AQP1 inhibitors may be a diuretics for treatment of renal diseases, whereas AQP1 induction might be applied to treat polyuria in humans in the further.

### Effect of aquaporin-1 gene knockout on nervous system

Even though swelling and shrinking in choroid plexus epithelium (CPE) in AQP1 null mice was remarkably slowed, AQP1 deletion did not affect choroid plexus size or structure and pressure-dependent cerebrospinal fluid (CSF) outflow [22,23]. AQP1-KO mice showed water influx into the CSF space virtually identical to WT mice [24]. Neither the Na^+^-dependent Cl^−^/HCO_3_^−^ exchanger (NCBE) nor Na^+^-K^+^-ATPase expression was affected in the CPE of AQP1-KO mice [25]. AQP1 deletion in mice produced a 5-fold reduction in CPE osmotic permeability, a 20–25% reduction in CSF production after forskolin administration, a significant reduction (56%) in intracranial pressure (ICP), a significant reduction (81%) in the central venous pressure (CVP), as well as an improved survival following focal cold-induced brain injury compared with WT mice [22,23]. AQP1 deficiency resulted in reduced baseline ventricular size, less ventricular dilation after kaolin injection, and a less severe ventriculomegaly than in WT mice [26]. Taken together, these results provided direct functional evidence for the involvement of AQP1 in CSF dynamics, suggesting AQP1 deletion might be protective in a brain trauma model [Table 1, Figure 1]. AQP1 inhibitors can be used for the therapy of human hydrocephalus and disorders of increased ICP from the important clinical enlightenment of the reduced ICP and CSF production in AQP1-KO mice.

Regarding the expression and function of AQP1 in the spinal cord, Oshio *et al.* [27,28] detected AQP1 expression in nociceptive neurons, and demonstrated that osmotic swelling in the superficial dorsal horn was reduced in AQP1-KO mice after exposure to hypotonic medium. AQP1-KO mice had reduced responsiveness to thermal and capsaicin chemical stimuli, although these mice displayed normal responses to noxious mechanical stimuli and the formalin test [27]. Also, Zhang *et al.* [29] found AQP1 deletion was associated with a reduced osmotic water permeability in freshly isolated dorsal root ganglia (DRG) neurons. Moreover, behavioral studies showed a greatly reduced thermal inflammatory pain perception in litter-matched AQP1-KO mice evoked by bradykinin, prostaglandin E_2_, and capsaicin as well as a reduced cold pain perception and distinct electrophysiological defects related to impaired Nav1.8. Na^+^ channel functioning in AQP1-deficient DRG neurons [Figure 1]. In addition, spontaneous and nerve growth factor-stimulated axonal extension was reduced in cultures of AQP1-deficient DRG neurons and DRG explants compared with the WT [30]. These data concerning the involvement of AQP1 in DRG axonal regeneration pointed to the dual role of AQP1 for DRG neurons in nociception and axonal growth. However, Shields *et al.* [31] reported that neither electrophysiological changes nor differences in nociceptive processing by complete behavioral analysis were shown in AQP1-KO mice [Table 1]. The mechanism by which AQP1 channel affecting nociceptor functions remains to be determined. Further investigations of this mechanism require extensive studies applying a battery of electrophysiological and behavioral tests in DRG neurons. AQP1 can be taken as a potential therapeutic target to accelerate neuronal regeneration.

### Effect of aquaporin-1 gene knockout on the lungs

AQP1-KO mice studies have already provided a substantial body of information about fluid movement between airspace, interstitial, and capillary compartments in the lungs. AQP1 deletion was associated with an 11-fold decrease in the lungs independent of the perfusate osmolyte size, a 1.4-fold decrease in the filtration coefficient in response to 5 cm H_2_O outflow pressure, and a great reduction in lung transcapillary osmotic water permeability [32]. AQP1 deletion also caused a moderate decrease in transcapillary water movement in response to hydrostatic pressure differences, a more than 2-fold reduction in lung water accumulation in response to a 5–10 cm H_2_O increase in pulmonary artery pressure for 5 min, and a 10-fold reduction in airspace-capillary water permeability, but did not affect active near-isosmolar alveolar fluid reabsorption [33] [Table 1]. These results suggest that AQP1 is important for physiological and pathological processes in the lungs.

However, the study by Song *et al.* [34] found that intraperitoneal thiourea infusion produced marked accumulation of lung water and formation of pleural effusions, but AQP1 deletion affected neither the amount of lung water accumulation nor the volume of the pleural effusions [34]. Despite its demonstrated role in epithelial and endothelial osmotic water permeabilities, AQP1 does not appear to play a significant role in active alveolar fluid clearance in the neonatal and adult lungs, or in the accumulation of pulmonary oedema in various types of acute lung injury [34]. Moreover, it was found that although aquaporins facilitated osmotically driven water transport in the airways, they played a minimal role in the processes of humidification of upper and lower airways, hydration of the airway surface liquid, and isosmolar fluid absorption in upper airways [35]. Similarly, although AQP1 expression is decreased in lipopolysaccharide (LPS)-induced acute lung injury in mice, depletion of AQP1 did not alter lung inflammation and lung edema induced by LPS, neither did AQP1 depletion affect lung edema formation or resolution, lung vascular permeability, or lung histology [36].

Overall, these studies suggest that AQP1 is not essential for the fluid movement in the peripheral lungs, for airspace humidification in the larger airways, or airway surface liquid fluid properties. This finding is in contrast to the proven role of AQP1 in kidney. Further experiments are required to define the exact role of AQP1 for lung functions under normal and pathological conditions.

### Effect of aquaporin-1 gene knockout on placental development and fetal growth

Transgenic AQP1-KO mice provided a unique model for determining the role of AQP1 for the placenta, fetal growth, and maternal–fetal fluid homeostasis. But the results are inconclusive [37–41].

Zheng *et al.* [37] found that AQP1-KO pregnant mice had a significantly lower number of embryos, lower fetal weight, and greater amount of amniotic fluid than WT. The AQP1-KO placenta demonstrated an increased degeneration with evidence of altered blood vessel structure and increased syncytiotrophoblast nodules [37]. A previous study by Mann *et al.* [38] also demonstrated an increase in the amount of more dilute amniotic fluid in AQP1-KO pregnant mice but there were no significant differences in fetal or placental weights compared with WT controls [38]. In contrast, the study by Guo *et al.* [39] showed that compared with AQP1^+/+^ mice, there was significant placental and embryonic overgrowth in AQP1^+/−^ (loss of maternal allele) mice and AQP1^−/−^ mice, but not in AQP1^+/−^ (loss of paternal allele) mice at embryonic day E12.5–E18.5 [Table 1]. AQP1 maternal deficiency resulted in increases in the placental mass and the labyrinthine layer area [39]. In addition, they performed the imprinting analysis of AQP1 and found that AQP1, as a novel imprinted gene, negatively correlated with the methylation status of the AQP1 promoter and exon 1 [39]. Taken together, these results showed that AQP1 indirectly influenced the embryonic development by affecting placental structure and functions.

Sha *et al.* [40] found that the osmotic water permeability in AQP1^−/−^ trophoblast cells was significantly lower than that in AQP1^+/+^ trophoblast cells in response to both hypotonic and hypertonic challenges [40]. Similar to this report, our study found that although no significant difference was observed in the amount of amniotic fluid among the AQP1 homozygote, heterozygote and WT mice at 13.5 gestational day (GD), AQP1 homozygote conceptus had a greater volume of amniotic fluid, lower osmolality, and calcium concentration than their WT counterparts at 16.5 GD. Loss of AQP1 expression in fetal membranes resulted in the down-regulation of AQP9 expression and up-regulation of AQP8 expression [41]. Our results confirmed that the loss of AQP1 expression in fetal membranes was related to increased amniotic fluid volume, and reduced osmolality (Table 1, Figure 1). We speculated that there might be mechanisms of mutual compensation among AQPs in their expression and functions in placentas and fetal membranes. Furthermore, our team is studying the mechanism of AQP1-KO affecting amniotic fluid volume and placental and embryo development, which will provide new ideas for the treatment of placenta-related diseases in humans.

### Effect of aquaporin-1 gene knockout on peritoneum

Peritoneal transport studies using AQP1-KO mice demonstrated that the osmotic water flux across the peritoneal membrane was mediated by AQP1. An early study by Yang *et al.* [42] showed that osmotically induced water movement was significantly reduced in AQP1^−/−^ mice compared with AQP1^+/+^ mice, which indicated that AQP1 provided an important route for osmotically driven water transport across the peritoneal barrier in peritoneal dialysis [Figure 1]. But spontaneous isosmolar fluid absorption from the peritoneal cavity was not affected by AQP1 deletion, which suggested that the AQP1 pathway had little clinical relevance to explain peritoneal fluid accumulation and reabsorption [42]. However, later studies [43] showed that AQP1^−/−^ mice had a complete loss of sodium sieving, a approximately 70% decrease in the initial and solute-free ultrafiltration (UF) and a 50% decrease in net UF, whereas glucose reabsorption from dialysate and dialysate-to-plasma osmolality were unchanged. Of note, the deletion of AQP1 had no effect on the structure of the peritoneal membrane, including the density or diameter of peritoneal capillaries [43]. As detailed above, studies in the AQP1-KO mice demonstrated the strict correlation between AQP1 abundance and solute-free water transport across the peritoneal membranes [Table 1, Figure 1].

To exclude the potential confounding phenotypic effects evoked by ubiquitous AQP1 deletion, using a Cre/loxP approach, Zhang *et al.* [44] generated and characterized a novel endothelial cell-specific and time-specific inducible AQP1-KO (AQP1^fl/fl^; Cdh5-Cre^+^) mouse model. They found that compared with controls, AQP1^fl/fl^; Cdh5-Cre^+^ mice showed no difference in basic biological parameters such as body weight and survival. During a 1-h miniperitoneal equilibration test, AQP1^fl/fl^; Cdh5-Cre^+^ mice exhibited much decreased indices for AQP1-related transcellular water transport (43.0% in net UF, 93.0% in sodium sieving, and 57.9% in free water transport), while the transport rates for small solutes of urea and glucose were not significantly altered [44]. Human future research is required to validate the essential role of endothelial AQP1 in UF and free water transport during peritoneal dialysis, which will provide a promising therapeutic target for preventing UF failure in peritoneal dialysis by regulation of the endothelial AQP1.

### Effect of aquaporin-1 gene knockout on eye

Several studies provided evidence for a novel role of AQP1 in the ocular physiology. Thiagarajah *et al.* [45] found that compared with WT mice, the corneal thickness was remarkably reduced in AQP1 null mice. AQP1 deletion reduced osmotic water permeability across the corneal endothelium and impaired the restoration of corneal transparency after experimental swelling. Wound healing and keratocyte appearance near the wound margin were significantly reduced in AQP1-KO mice. Neutrophils were more abundant in corneas and corneal epithelial wound repair was delayed in AQP1-null mice by 24 h after injury [45]. *In vitro* and *in vivo* observations indicated that AQP1 deletion reduced the migration of keratocyte. The slowed healing of stromal wounds under AQP1 deficiency may be a consequence of impaired keratocyte migration [46]. Taken together, the marked impairment of corneal recovery in AQP1 null mice has potentially important implications for the treatment of corneal edema, by which will reduce complications of eye surgery [Table 1].

In addition, although AQP1 deficiency did not alter the lens morphology or transparency, Ruiz-Ederra *et al.* [47] found a remarkably accelerated cataractogenesis in both *in vitro* and *in vivo* models of cataract formation [47]. Thus, AQP1 facilitates the maintenance of transparency in lens and opposes cataract formation [Table 1, Figure 1]. However, oxygen-induced retinal microvessel proliferation was not affected by AQP1 deletion in a neonatal mouse model of oxygen-deprivation retinopathy, which suggested that AQP1 inhibitors may be used to treat ocular disorders such as glaucoma because retinal vessel proliferation was AQP1 independent [48]. In conclusion, as a new determinant of ocular function, modulation of AQP1 might be applied to treat different human eye diseases in the future.

### Effect of aquaporin-1 gene knockout on gland secretion

Several groups have reported different findings regarding the effect of AQP1-KO on gland secretion. Analysis of anterior pituitaries from AQP1-KO mice showed a reduced prohormone convertase 1 and 3, carboxypeptidase E, and ACTH levels in the pituitary and serum compared with WT mice. In corroboration with these observations, down-regulation of AQP1 expression in the AtT20 cells resulted in attenuated regulated secretion of ACTH, a dramatic loss of dense-core secretory granule (DCSG) proteins, and decreased DCSG biogenesis [49] [Table 1]. These findings demonstrated that AQP1 was important for maintaining secretory function and granule biogenesis, and hence normal hormone sequestration in endocrine cells. However, neither deletion of AQP1 in the salivary microvascular endothelia affected saliva production [50] nor deletion of AQP1 in mice affected pancreatic secretion [51] and basal or pilocarpine-stimulated tear fluid production or chloride concentration [52] as well as tear film pH. But AQP1 deficiency appeared to be associated with reduced tear film (K^+^) [53]. Further studies need to be performed to characterize the role of AQP1 in gland secretion and their potential as novel drug targets to treat endocrine tumors.

### Effect of aquaporin-1 gene knockout on cardiovascular system

Several studies have characterized the AQP1-KO cardiovascular phenotypes [54–57]. Compared with age-matched WT littermate, AQP1-KO mice exhibited a marked microcardia, decreased myocyte transverse dimensions and a significant decrease in the thickness of the arterial walls both in the aorta and mesenteric artery from both genders, whereas no change in the capillary density [54]. Both male and female AQP1-KO mice had lower systolic and mean blood pressure but normal diastolic blood pressure with preservation of the circadian variation, which mainly occurred during the night [54]. Interestingly, the lower mean BP was not attributable to altered water balance or autonomic dysfunction because AQP1-KO mice exhibited a normal baro reflex response, with a reduction in heart rate proportional to vasoconstrictor-induced increase in blood pressure, and a slope identical to WT litter-mates [Figure 1]. AQP1-KO mice showed an unchanged NO-dependent relaxation in aortic and mesenteric rings, but a potentiation of the prostanoids-dependent relaxation together with increased expression of COX-2. In addition, AQP1 genetic deletion was accompanied by a compensatory up-regulation of AQP4, AQP7, and AQP8 in the heart and AQP7 in the aorta [54] [Table 1]. These findings identified new roles of AQP1 in cardiovascular homeostasis. Moreover, AQP1 deficiency augmented lesion development in angiotensin II-promoted atherosclerosis. Thus, the normal function of AQP1 may afford a cardiovascular protection [55] [Table 1].

However, the study performed by Al-Samir *et al.* [56] observed major morphological alterations in the AQP1-deficient heart including a reduced cardiac muscle mass, reduced left ventricles (LV) wall thickness, reduced cross-sectional area of ventricular myofibers, reduced ratio of capillaries to myofibers, and reduced absolute density of capillaries in LV tissue. Surprisingly, anesthetized KO mice, even under dobutamine stimulation, exhibited an entirely normal cardiac function [56]. Moreover, they found that AQP1 deficiency limited the maximal oxygen consumption under normoxic or hypoxic conditions due to the reduced cardiac muscle mass and wall thickness [57]. Based on these observations, a reduced maximal cardiac output was postulated, but AQP1 deficiency did not appear to affect arterial oxygen saturation as well as respiratory rate [57]. Future studies are needed to verify the specific effect of AQP1 deletion in pathological situations such as ischemia/reperfusion and pressure overload. In view of potential new roles of AQP1 in cardiovascular homeostasis, agonists and antagonists of AQP1 are being developed their use in cardiovascular diseases associated with heart and vessel remodeling, like treating hypertrophic cardiac remodeling or microcardia.

### Effect of aquaporin-1 gene knockout on digestive system

Several laboratories reported association of AQP1 deletion with developmental or acquired structural abnormalities in gastrointestinal and hepatobiliary function. Ma *et al.* [58] found that the AQP1-deficient mice given a high-fat diet gained remarkably less weight than matched WT mice [58]. The AQP1-deficient mice acquired an oily appearance, manifested serum hypotriglyceridemia, and developed steatorrhea with increased stool triglyceride content. Lipase activity in feces and small intestine in the young mice was remarkably greater in AQP1 null than WT mice on low- and high-fat diets, while absorption of (^14^C) oleic acid from small intestine was not affected by AQP1 deletion. Moreover, older mice that are less sensitive to high-fat diet showed a 3-fold increase of pancreatic fluid flow in response to secretin/cholecystokinin stimulation, but volumes, pH, and amylase activities were not significantly altered by AQP1 deletion, nor were bile flow rates and bile salt concentrations [58]. Together, these results established a dietary fat metabolism defect in AQP1 null mice, particularly in young mice [Table 1]. The data from the AQP1-KO mouse would suggest that these findings can be extended to our human. Possible involvement of AQP1 as novel drug targets might be treat malnutrition or obesity.

In addition, although AQP1 deletion had a 10-fold reduced apical plasma membrane water permeability in gallbladder, it affected neither gallbladder size and morphology nor the bile osmolality and bile salt concentration, suggesting that, despite its role in transcellular water transport, the impact of AQP1 deletion on gallbladder functions is limited [59]. Nevertheless, after bile duct ligation AQP1-KO mice have reduced angiogenesis, reduced fibrosis, and less portal hypertension, suggesting a prominent role for AQP1 in the pathological changes often observed during chronic liver diseases. AQP1 could be a molecular target for the treatment of chronic liver disease [60] [Figure 1].

### Effect of aquaporin-1 gene knockout on cell migration

Most findings support a fundamental role of AQP1 in cell migration, which is central to diverse biological phenomena including angiogenesis, wound healing, organ regeneration, and tumor spreading. Saadoun *et al.* [61] found that targeted AQP1 gene disruption in mice reduced angiogenesis *in vivo* [61] [Table 1]. Although the proliferation and adhesion of aortic endothelial primary cultures AQP1-null mice appeared to be similar to those from WT mice, cell migration was impaired in AQP1-deficient cells, with abnormal vessel formation *in vitro*. Impaired tumor growth in AQP1-null mice, including a reduced tumor vascularity and extensive tumor necrosis, but an enhanced survival of tumor-bearing mice was observed [60] [Figure 1]. Likewise, AQP1 deletion did not affect the chondrocyte proliferation rate, but the serum-induced transwell migration rate and adhesion of AQP1-deficient chondrocytes to type II collagen, as well as plasma membrane water permeability were reduced compared with WT chondrocytes [62]. Therefore, a proper level of AQP1 expression in chondrocytes may be required for graft formation and transplantation. The activity and molecular pathways by which AQP1 affects endothelial cell function and chondrogenesis remain to be characterized.

It was reported that in the AQP1-deficient mice, the volume, vessel density, and lung metastasis of polyoma formed by the mouse mammary tumor virus middle T oncogene were all reduced. These results had implicated AQP1 in tumor development, and leading to postulation of AQP1 as a potential target for adjuvant therapy of solid tumors [63]. In addition, using peritoneal macrophages isolated from AQP1-deficient mice, Tyteca *et al.* [64] found that ablation of AQP1 caused macrophage elongation, axial polarization, and membrane lipid orientation to the leading edge. AQP1 ablation affected the macrophage morphology, cytoskeletal organization, membrane polarization, and migration. The peritoneal infiltration was attenuated in AQP1^−/−^ mice, with a 2-fold decreased number of infiltrating macrophages [64] [Table 1]. Taken together, these results indicated appropriate expression of AQP1 was required for normal function of macrophages and potentially other cell lineages important for tissue remodeling and wound healing [Figure 1]. In further human studies, pharmacological inhibition of AQP1 may have utility in cancer therapy, whereas AQP1 induction might accelerate wound healing and facilitate organ regeneration.

## Conclusions

In summary, AQP1 is a water-selective transporting protein affecting the water permeability of cell membranes. The phenotype analysis of AQP1-KO mice in the placenta, kidney, lung, heart, brain, peritoneum, gland, eye, and gastrointestins and liver supports a paradigm that AQP1 can facilitate near-isosmolar transepithelial fluid absorption/secretion as well as rapid vectorial water movement driven by osmotic gradients. Since water absorption, transportation, secretion, and maintenance of normal osmolality represents a fundamental part of physiology, depending on cell types and tissues/organs, this direct action of AQP1 contributes to a diverse set of functions such as glomerular filtration and urinary concentrating efficiency in the kidney, CSF homeostasis, placenta and embryo development, water flux across the peritoneal membrane, systolic and mean blood pressure. Paradoxically, some manifestations in the AQP1-KO mice, e.g., hypotriglyceridemia, angiogenesis deficiency, and changed behavior of tumor, appeared to have no or little connection to water homeostasis at the first sight, and the mechanisms/pathways involved are far from current understanding. Importantly, the observed defects in AQP1-KO mice may provide study or treatment models for human diseases. The data obtained from AQP-KO mice studies suggest AQP1 inhibitors may have clinical indications as diuretics and the glaucoma, cerebral edema, elevated intraocular pressure, malignancies, and other conditions directly or indirectly related to abnormal fluid homeostasis, whereas AQP1 induction might be applied to treat polyuria, polyhydramnios, cataract, to accelerate wound healing, or to facilitate organ regeneration. Although current knowledge is mostly limited to AQP1, the best studies prototype of AQPs, the expression patterns and activities of other AQPs in various tissues deserve a thorough investigation. Further studies are also needed to explore the mechanisms and pathways initiated by AQPs, the functional interactions and mutual compensations among AQPs, and the regulation of AQP expression along organogenesis and embryo development.

## Abbreviations

AQP1: aquaporin 1
AQP1-KO: AQP1 knockout
CPE: choroid plexus epithelium
CSF: cerebrospinal fluid
DCSG: dense-core secretory granule
DRG: dorsal root ganglia
GD: gestational day
GFR: glomerular filtration rate
ICP: intracranial pressure
IMCD: inner medullary collecting duct
LPS: lipopolysaccharide
LV: left ventricle
SNGFR: single nephron GFR
TDLH: thin descending limb of Henle
UF: ultrafiltration
V2R: vasopressin type 2 receptor
WT: wild-type
